# Recruitment of Polysynaptic Connections Underlies Functional Recovery of a Neural Circuit after Lesion

**DOI:** 10.1523/ENEURO.0056-16.2016

**Published:** 2016-08-26

**Authors:** Akira Sakurai, Arianna N. Tamvacakis, Paul S. Katz

**Affiliations:** Neuroscience Institute, Georgia State University, Atlanta, Georgia 30302-5030

**Keywords:** brain injury, central pattern generator, functional recovery, mollusk, network reorganization, synapse

## Abstract

The recruitment of additional neurons to neural circuits often occurs in accordance with changing functional demands. Here we found that synaptic recruitment plays a key role in functional recovery after neural injury. Disconnection of a brain commissure in the nudibranch mollusc, *Tritonia diomedea*, impairs swimming behavior by eliminating particular synapses in the central pattern generator (CPG) underlying the rhythmic swim motor pattern. However, the CPG functionally recovers within a day after the lesion. The strength of a spared inhibitory synapse within the CPG from Cerebral Neuron 2 (C2) to Ventral Swim Interneuron B (VSI) determines the level of impairment caused by the lesion, which varies among individuals. In addition to this direct synaptic connection, there are polysynaptic connections from C2 and Dorsal Swim Interneurons to VSI that provide indirect excitatory drive but play only minor roles under normal conditions. After disconnecting the pedal commissure (Pedal Nerve 6), the recruitment of polysynaptic excitation became a major source of the excitatory drive to VSI. Moreover, the amount of polysynaptic recruitment, which changed over time, differed among individuals and correlated with the degree of recovery of the swim motor pattern. Thus, functional recovery was mediated by an increase in the magnitude of polysynaptic excitatory drive, compensating for the loss of direct excitation. Since the degree of susceptibility to injury corresponds to existing individual variation in the C2 to VSI synapse, the recovery relied upon the extent to which the network reorganized to incorporate additional synapses.

## Significance Statement

In cases of permanent neuronal injury, functional recovery can occur through the reorganization of the remaining neural circuitry. Here, this study shows that a molluscan neural circuit recruits additional neurons in response to a lesion and that the extent of recruitment predicts the extent of behavioral recovery. Interestingly, the initial susceptibility of the circuit to this lesion reflects the strength of a specific synapse within the circuit, but the functional recovery correlates with polysynaptic recruitment from outside the canonical motor circuit. Thus, even in a well defined invertebrate neural circuit, there are indirect, polysynaptic pathways that provide compensatory function or flexibility to the circuit. Such individual variability appears to be hidden under normal conditions but becomes relevant when challenged by neural injury.

## Introduction

Understanding the mechanisms underlying the recovery of function after brain injury is hampered by variability among individuals and the complexity of neural circuits. Individuals differ from one another to such an extent that it can be difficult to predict outcomes in cases of traumatic brain injury (Lingsma et al., 2010) or stroke (Cramer, 2008a,b). There are many reports of neural circuit reorganization underlying behavioral recovery after the loss of neural function due to nervous system injury (Barbeau et al., 1999; Antri et al., 2002; Navarro et al., 2007; Cramer, 2008a; Kumru et al., 2010; Martinez et al., 2011; Ueno et al., 2012; Martinez and Rossignol, 2013; Filli and Schwab, 2015). More tractable invertebrate nervous systems can provide insight into the problems of individual variability and recovery from injury.

**Figure 1.**
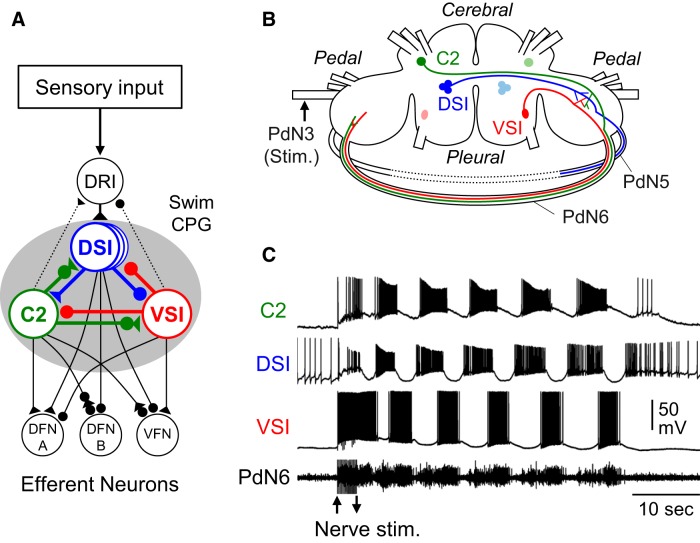
The *Tritonia* swim central pattern generator. ***A***, The neural circuit underlying the swim motor pattern. The shaded oval indicates the neurons that comprise the swim CPG. The CPG consists of the following three types of interneurons: C2, DSIs, and VSI. The DSIs receive excitatory drive from the dorsal ramp interneuron (DRI), which serves as a gating neuron for the CPG (Frost and Katz, 1996). Through DRI, C2, and VSI make indirect synaptic actions onto DSI. All CPG neurons are electrically coupled to contralateral counterparts that are not represented here. Filled triangles represent excitatory synapses, and filled circles represent inhibitory synapses. Combinations of triangle and circle are multicomponent synapses. Dotted lines indicate that the connection is either polysynaptic or not determined. DFN-A, -B, dorsal flexion neurons A and B; VFN, ventral flexion neuron. Based on Getting et al. (1980), Getting (1981), Hume and Getting (1982), and Frost and Katz (1996). ***B***, Schematic drawing of a dorsal view of the *Tritonia* brain, showing the locations of the swim CPG neurons and their axonal projections. DSIs and C2 are located on the dorsal surface of the cerebral ganglion. VSI is located on the ventral side of the pleural ganglion. All neurons in the swim CPG are bilaterally arranged. C2 and VSI project their axons through PdN6, whereas DSI projects through PdN5. PdN5 was removed upon isolation of the brain, as indicated by dotted lines. ***C***, An example of the swim motor pattern recorded intracellularly from the three CPG neurons and extracellularly from PdN6 in an isolated brain preparation. The bursting pattern was elicited by stimulation of the left body wall nerve, PdN3, using voltage pulses. Arrows show onset and offset of the nerve stimulation.

In the brain of the nudibranch mollusc, *Tritonia diomedea* (also called *Tritonia tetraquetra*, by Pallas in 1788), disconnecting one of the commissures impairs the rhythmic swimming behavior of the animal and the motor pattern underlying it (Sakurai and Katz, 2009). The central pattern generator (CPG) circuit for the *Tritonia* swimming behavior consists of the following three bilaterally represented neuronal types: Dorsal Swim Interneuron (DSI), Cerebral Neuron 2 (C2), and Ventral Swim Interneuron (VSI; Fig. 1*A*,*B*), which form a network oscillator. Through their synaptic interactions and membrane properties, these neurons produce the rhythmic motor pattern that drives the swimming movements (Fig. 1*C*; Getting, 1989b). The sequence of activity is that DSI excites C2, which is then coactive with DSI (Katz, 2007, 2009). C2 excites VSI, and then VSI inhibits DSI and C2, ending the cycle. C2 and VSI both send axons through one of the pedal commissures, Pedal Nerve 6 (PdN6), which connects the two pedal ganglia (Fig. 1*B*). C2-evoked excitation of VSI is critical for the generation of the swim motor pattern (Calin-Jageman et al., 2007). The C2-to-VSI connection in the distal pedal ganglion plays a dominant role in evoking the C2-evoked excitation of VSI; which consequently causes antidromic spikes travelling through PdN6 (see Fig. 6*A*). Severing their axons in PdN6 impairs motor pattern production, but the system spontaneously recovers over the course of a few hours to a day (Sakurai and Katz, 2009).

In this study, we found that enhanced recruitment of polysynaptic excitation appears to underlie the functional recovery of the impaired CPG by compensating for the loss of excitation from C2 to VSI after the lesion. These polysynaptic connections play little or no role in generating the motor pattern under normal conditions (Sakurai et al., 2014). However, they increase in strength and become functionally relevant to the recovery of the swim motor pattern. Thus, even in the well defined CPG of an invertebrate, there are indirect, polysynaptic pathways between neurons that appear to provide a compensatory function or flexibility to the neural circuit.

## Materials and Methods

### Animal surgery

Specimens of *T. diomedea*, which is a hermaphrodite animal, were obtained from Living Elements Ltd. Before the surgery for the commissure lesion, a pair of animals (test animal and sham animal) were placed in ice-chilled artificial seawater (Instant Ocean Sea Salt for Aquariums, Instant Ocean) containing 0.1% 1-phenoxy-2-propanol (Redondo and Murray, 2005; Wyeth et al., 2009) for 1 h. Then, a small incision was made in the skin behind the rhinophores to expose the brain. In the experimental animals, one of the pedal commissures (PdN6; Willows et al., 1973) was cut near the right pedal ganglion with fine scissors; whereas in the sham controls, PdN6 was exposed but not cut. The skin incision was stitched with silk thread and sealed with cyanoacrylate glue (Ethyl Cyanoacrylate, WPI).

Animals with lesions were paired with sham-operated animals for the behavioral assay. The observer was blind to the condition of each animal in a trial. The swim was induced by applying 0.5 ml of a 5 m NaCl solution to the dorsal body surface. The number of body flexions was defined as the number of complete ventrally directed body flexions. Retraction of the body without a body flexion in response to the stimulus was considered as a swim failure and counted as zero flexions. The number of body flexions during the escape behavior was measured at 28, 24, 16, 12, and 2 h prior to the commissure transection and at 2, 6, 10, and 19 h after the surgery.

### Isolated brain preparations

Before dissection, the animal was chilled to 4°C in the refrigerator. The brain was removed from the animal and pinned to the bottom of a Sylgard-lined dish, and constantly superfused with saline at 4°C. Physiological saline solution composition was as follows (in mm): 420 NaCl, 10 KCl, 10 CaCl_2_, 50 MgCl_2_, 11 d-glucose, and 10 HEPES, pH 7.6. The cell bodies of the neurons were exposed by removing the connective tissue sheath from the surface of the ganglia (Willows et al., 1973). Left PdN3 was introduced into a suction electrode made from polyethylene tubing for electrical stimulation that evokes the swim motor pattern. PdN6 was sucked into a suction electrode fabricated from a pulled, fire-polished, borosilicate glass tube (inner diameter, 1.0 mm; outer diameter, 1.5 mm) in the en passant configuration. The preparation was left for at least 3 h and superfused in saline at 10°C before the electrophysiological experiments. For all experiments, the ganglia were superfused at 2 ml/min at 10°C.

The swim interneurons were identified by soma location, electrophysiological monitoring of axonal projection (Fig. 1*B*), coloration, synaptic connectivity, and activity pattern at rest and during the swim motor program, as previously described (Getting, 1981, 1983). There are the following three types of CPG neurons: DSIs, C2, and VSI-B. For simplicity, we will refer to VSI-B as VSI in this article. C2 and DSI have cell bodies on the dorsal surface of the cerebral ganglion and project their axons toward the contralateral pedal ganglion; whereas, VSI has its cell body on the ventral side of the pleural ganglion and projects its axon toward the ipsilateral pedal ganglion (Fig. 1*B*). To record from both C2 and VSI, the brain was twisted around the cerebral commissure as described by Getting (1983). The experiment was discarded if one of the neurons died during the recordings.

The swim motor program was evoked by stimulating the left PdN3 with a train of voltage pulses (5–15 V, 1.5 ms) at 5 Hz for 3 s via a suction electrode. Unilateral electrical stimulation of PdN3 is sufficient to elicit the bilaterally symmetric swim motor pattern (Fig. 1*C*). Electrical stimuli were given at intervals of >10 min to avoid habituation of the swim motor pattern (Frost et al., 1996).

In the isolated brain preparation, PdN6 was functionally disconnected by either physical transection or by blocking action potential propagation in PdN6 by the local application of tetrodotoxin (10^−4^ m; Sigma-Aldrich) in a suction pipette that contained the commissure. It was previously shown that there was no statistical difference between cutting and pharmacological disconnection (Sakurai and Katz, 2009; Sakurai et al., 2014). Physical transection of PdN6 induces a brief barrage of action potentials in VSI. The responses varied among preparations; however, the injury firing never exceeded 2 min (Sakurai and Katz, 2009; Sakurai et al., 2014). It was shown previously that physical transection has no long-term effect on the resting membrane potential or spontaneous spiking activities in the CPG neurons (Sakurai and Katz, 2009). In this study, both procedures were referred to as “PdN6 disconnection” or “cut.”

In some experiments, the bathing medium was switched to a high-divalent cation (Hi-Di) saline containing five times the normal concentration of divalent cations, which raises the threshold for spiking and reduces spontaneous neural firing. The composition of the Hi-Di saline was (in mm): 285 NaCl, 10 KCl, 25 CaCl2, 125 MgCl2, 10 d-glucose, and 10 HEPES, pH 7.4.

### Electrophysiological recordings and stimulations

Neurons were impaled with glass microelectrodes filled with 3 m potassium chloride (12–44 MΩ). To test C2-evoked synapses, C2 was made to fire at 10 Hz using repeated injection of 20 ms current pulses that each evoked a single action potential. Axoclamp-2B amplifiers (Molecular Devices) were used for all electrophysiological experiments. Recordings were digitized at 2–6 kHz with a 1401plus analog-to-digital converter from Cambridge Electronic Design (CED). Data acquisition and analysis were performed with Spike2 software (CED) and SigmaPlot (Jandel Scientific). The resting potential for each neuron was measured within 10 min after electrode impalement or upon removal of the electrode from the neuron.

In isolated brain preparations, the number of swim cycles, which corresponds to body flexions in the intact animal, was defined as the number of VSI bursts during the swim motor pattern. A cluster of two or more action potentials with intervals of <1 s was considered as a burst. VSI often exhibited a few spikes during nerve stimulation; these were not counted as a burst. The number of intraburst spikes in VSI was measured from the second burst in the swim motor pattern because after the lesion the spike number decreased more markedly in later cycles.

To quantify the polysynaptic action of C2/DSI onto VSI, the frequency (in counts per second) of recruited EPSPs in VSI was measured during a 6 s window after the end of the stimulation. Care was taken not to include stimulus artifacts. EPSPs <0.1 mV were excluded from the analysis, because it is difficult to distinguish them from stimulus artifacts. No polysynaptic IPSPs were seen in VSI when C2/DSI was stimulated. We did not distinguish between electrical and chemical synapses in this study. To measure the direct synaptic action of C2/DSI onto VSI, Hi-Di saline was used to remove polysynaptic input. The amplitude of depolarization was measured from the basal resting potential to the maximal peak; whereas, the amplitude of hyperpolarization was measured from the peak of the preceding depolarization, if there was one, to the subsequent trough.

### Statistics

We define “impairment” as a decrease in the number of swim cycles after the lesion and “recovery” as the increase in the number of swim cycles the next day from the impaired state. Statistical comparisons were performed using SigmaPlot version 12.5 (Jandel Scientific) for Student’s *t* test (one-tailed), Pearson product moment correlation, linear regression, Levene median test, one-way ANOVA, one-way repeated-measures ANOVA followed by all pairwise multiple comparison (Holm–Sidak method), and Kruskal–Wallis one-way ANOVA on ranks with Dunn’s method (Table 1). The Shapiro–Wilk test was used to assume the normality of the data structure. In all cases, *p* < 0.05 was considered to be significant. Coefficient of variance (CoV) was calculated by dividing the SD by the mean. The results are expressed as the mean ± SD.

## Results

### Recovery of swimming behavior varied across individuals after commissure disconnection

**Figure 2.**
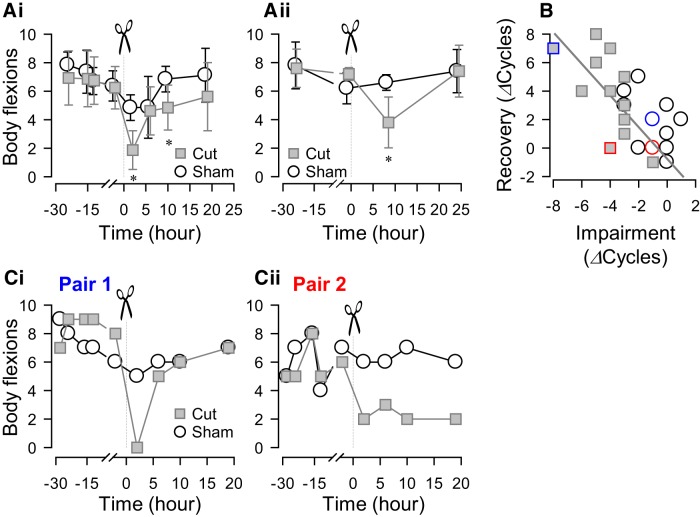
Individual variability in recovery of swimming after a lesion. ***A***, Changes in the number of body flexions during the escape swim behavior for animals with PdN6 transected (gray squares) and sham-operated controls (white circles) measured over time. PdN6-transected animals were paired with sham-operated animals and tested in a blind fashion. In one set of experiments, the swim behavior was evoked four times (24, 15, 12, and 2 h) prior to the transection and four times (2, 6, 10, and 19 h) after the transection (*N* = 8 for both cut and sham; ***Ai***). In the other set, the swim was evoked twice (24 and 1 h) prior to the transection and twice (8 and 24 h) after the transection (*N* = 4 for both cut and sham; ***Aii***). A two-way repeated-measures ANOVA was conducted for each graph to test statistical significance between transected and sham animals. For both intervals, there was no statistical significance between transected and sham animals (***Ai***: *F*_(1,93)_ = 3.24, *p* = 0.09; ***Aii***: *F*_(1,24)_ = 1.48, *p* = 0.26). However, pairwise multiple comparison procedures (Holm–Sidak method) revealed a significant difference (*) between sham and tested animals at 2 and 10 h in ***Ai*** (*p* = 0.002 and 0.04, respectively), and at 8 h in ***Aii*** (*p* = 0.003). ***B***, The increase in the number of body flexions upon recovery showed a significant correlation, with the decrease caused by PdN6 disconnection (impairment) in test animals (gray squares; *R* ^2^ = 0.46, *p* = 0.01 by Pearson product moment correlation, *N* = 13), but not in sham animals (open circles; *p* = 0.09 by Pearson product moment correlation, *N* = 13). Blue and red symbols correspond to the examples in ***C*** (blue, pair 1; red, pair 2). ***C***, Two examples (pair 1 and pair 2) showing different effects on the number of body flexions during the escape swim behavior for animals in response to PdN6 transection (gray squares) compared with sham-operated controls (white circles). In one example, cutting PdN6 caused a large decrease in the number of body flexions compared with sham, which was followed by recovery (pair 1, ***Ci***). In the other example, the decreased number of body flexions did not recover after 20 h (pair 2, ***Cii***).

Disconnection of PdN6 often reduces the number of the body flexions with a significant variation among individuals (Sakurai et al., 2014); however, the reduced number of body flexions increases by the next day (Sakurai and Katz, 2009). Here we found that the recovery from behavioral impairment was also variable among individuals (Fig. 2).

**Table 1:** Statistical table

Figure	Panel	Date structure	Test type	*p* value	*N*
2	*Ai*	Normally distributed	Two-way RM ANOVA	0.09	8,8
2	*Ai*	Normally distributed	Pairwise multiple comparison (Holm–Sidak method)	0.002, 0.04	8, 8
2	*Aii*	Normally distributed	Two-way RM ANOVA	0.26	4, 4
2	*Aii*	Normally distributed	Pairwise multiple comparison (Holm–Sidak method)	0.003	4, 4
2	*C*	Normally distributed	Pearson product moment correlation	0.01, 0.09	12, 12
3	*B*	Normally distributed	One-way RM ANOVA with Holm–Sidak method	<0.001	3–21
3	*C*	Normally distributed	One-way RM ANOVA with Holm–Sidak method	<0.001	5–28
4	*Ai*	Normally distributed	Pearson product moment correlation	0.86	75
4	*Aii*	Normally distributed	Pearson product moment correlation	0.54	73
4	*Aiii*	Normally distributed	Pearson product moment correlation	0.84	45
4	*Aiv*	Normally distributed	One-way ANOVA	0.59	75, 73, 45
4	*Bi*	Normally distributed	Pearson product moment correlation	0.54	67
4	*Bii*	Normally distributed	Pearson product moment correlation	0.45	68
4	*Biii*	Normally distributed	Pearson product moment correlation	0.85	44
4	*Biv*	Normally distributed	One-way ANOVA	0.97	67, 68, 44
4	*Ci*	Normally distributed	Pearson product moment correlation	0.36	89
4	*Cii*	Normally distributed	Pearson product moment correlation	0.76	84
4	*Ciii*	Normally distributed	Pearson product moment correlation	0.31	54
4	*Civ*	Normally distributed	One-way ANOVA	0.61	92, 88, 57
5	*A*	Normally distributed	Pearson product moment correlation	0.13	54
5	*B*	Normally distributed	Pearson product moment correlation	<0.001	54
8	*Aiii*	Normally distributed	Linear regression	0.008	33
8	*Aiv*	Normally distributed	Linear regression	0.99	33
8	*Biii*	Normally distributed	Linear regression	0.10	25
8	*Biv*	Normally distributed	Linear regression	0.94	25
8	*Ciii*	Normally distributed	Linear regression	<0.001	30
8	*Civ*	Normally distributed	Linear regression	0.93	30
9	*Aiii*	Normally distributed	Linear regression	0.43	18
9	*Aiv*	Normally distributed	Linear regression	0.44	18
9	*Av*	Normally distributed	Linear regression	0.49	18
9	*Avi*	Normally distributed	Linear regression	0.17	18
9	*Biii*	Normally distributed	Linear regression	0.99	13
9	*Biv*	Normally distributed	Linear regression	0.57	13
9	*Bv*	Normally distributed	Linear regression	0.11	13
9	*Bvi*	Normally distributed	Linear regression	0.59	13
9	*Ciii*	Normally distributed	Linear regression	0.51	13
9	*Civ*	Normally distributed	Linear regression	0.57	13
9	*Cv*	Normally distributed	Linear regression	0.06	13
9	*Cvi*	Normally distributed	Linear regression	0.77	13
10	*Aiii*	Normally distributed	Linear regression	0.04	28
10	*Biii*	Normally distributed	Linear regression	0.11	18
10	*Ciii*	Normally distributed	Linear regression	0.002	23
11	*B*	Normally distributed	One-way ANOVA	0.38	41, 39, 30
11	*C*	Normally distributed	One-way ANOVA	0.18	45, 43, 36
11	*D*	Normality not assumed	Kruskal–Wallis one-way ANOVA on ranks with Dunn’s method	<0.001	45, 47, 41
11	*G*	Normality not assumed	Levene median test	<0.02, <0.002	47, 41
11	*Hi*	Normally distributed	Linear regression	0.78	14
11	*Hii*	Normally distributed	Linear regression	0.57	14
11	*I*	Normally distributed	Linear regression	0.004	27
11	*J*	Normally distributed	Linear regression	<0.001	20

RM, Repeated measures.

Figure 2*A* shows the time course over which the number of body flexions changed before and after the PdN6 disconnection test at two different test intervals. In the first experiment (Fig. 2*Ai*), the swim was evoked three times at 4 h intervals from 2 h after PdN6 disconnection (cut), and then once the next day (19 h after cut). In the second experiment (Fig. 2*Aii*), the swimming behavior was tested at 8 and 24 h after the cut. In both experiments, the lesioned animals showed a significant decrease in the number of body flexions after the surgery, which recovered by 19–24 h (Fig. 2*A*). Within 24 h after PdN6 disconnection, 88% of animals (11 of 13 animals) showed an increase in the number of body flexions to varying degrees. Because of this, there is no statistical difference in the mean values between sham and cut animals in both sets of experiments.

The change in the number of body flexions 19–24 h after the cut showed a significant correlation with the cut-induced reduction (Fig. 2*B*, gray squares with a gray linear regression line). In contrast, sham animals were less affected by the surgery, and the correlation was not significant (Fig. 2*B*, open circles). This indicates that the gradual increase in the number of swim cycles after the surgery was due to recovery from an impaired state rather than an overall increase in the number of body flexions. On average, both the impairment and the extent of recovery in cut animals (−4 ± 1.7 and 3.7 ± 2.8 cycles, respectively, *N* = 13) were significantly greater than those in sham animals (−1 ± 1.4 and 2.2 ± 1.9 cycles, respectively, *N* = 13; *p* < 0.001 by Student’s *t* test).

There was remarkable individual variability in the extent to which the swim behavior recovered from the impaired state (Fig. 2*C*). Figure 2*Ci*shows an example of an animal whose swimming behavior was completely disrupted by PdN6 disconnection, but who recovered back to five flexions in 5 h and seven flexions in 19 h. However, a small proportion of animals (2 of 17 animals) exhibited no recovery at all after the PdN6 lesion, as shown in Figure 2*Cii*. The changes in the number of body flexions upon recovery ranged from −1 to 8, whereas those in the sham-operated animal ranged from −1 to 5 cycles. The cut group exhibited a greater variance in the change in the number of body flexions after injury, relative to before the injury, than did the sham-operated group (*p* < 0.05, by Levene median test; *N* = 13).

### Recovery of the swim motor pattern varied across individuals after commissure disconnection

**Figure 3.**
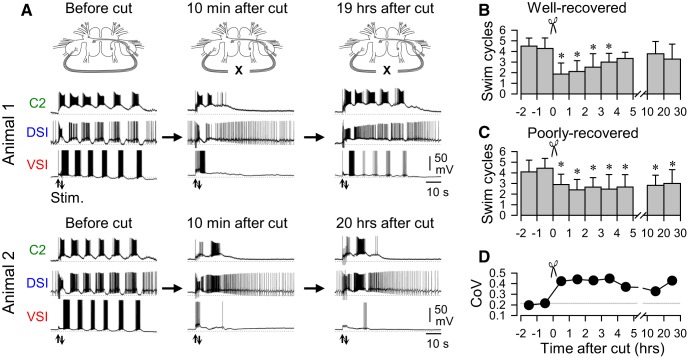
Individuals differed in the extent of motor pattern recovery after disconnection of PdN6. ***A***, Simultaneous intracellular recordings from C2, DSI, and VSI from two representative animals (animals 1 and 2). PdN6 was transected as indicated by inset drawings above the traces. The stimulus was delivered to the left PdN3 to trigger the swim motor pattern. Arrows (Stim) indicate the time of PdN3 stimulation. The brains from both animals 1 and 2 showed a decrease in the number of VSI bursts after cutting PdN6 (from 6 to 1). The number of swim cycles recovered back to five bursts in the brain from animal 1 at 19 h after PdN6 cut, whereas it remained impaired in the brain from animal 2. A dotted line on each trace indicates −50 mV membrane potential. ***B***, Bar graph showing averages of the swim cycles binned at 1 h intervals after PdN6 transection from well recovered preparations that exhibited ≥25% recovery in the number of swim cycles. Each bar shows the mean ± SD across preparations. Asterisks indicate significant difference from control (−1 to 0 h; *p* < 0.005 by one-way repeated-measures ANOVA with Holm–Sidak method). ***C***, Bar graph showing averages of the swim cycles at binned at 1 h intervals after PdN6 disconnection from poorly recovered preparations that showed <25% recovery in the number of swim cycles. Asterisks indicate significant difference from control (−1 to 0 h; *p* < 0.001 by one-way repeated-measures ANOVA with Holm–Sidak method). ***D***, The CoV of the number of bursts increased after PdN6 disconnection for all preparations.

It was previously shown in isolated brains that there was a wide variation among individuals in the extent of impairment of the swim motor pattern upon PdN6 disconnection (Sakurai and Katz, 2009). Similar individual variability was also seen in the extent of recovery (Fig. 3). Figure 3*A* shows examples of swim motor patterns recorded from two different preparations (animal 1 and animal 2), which consisted of six cycles of alternating bursts in the swim interneurons before PdN6 disconnection (before cut). After disconnecting PdN6 (10 min after cut), the motor pattern was “impaired” in both preparations (i.e., the number of VSI bursts was reduced). The next day (19 h after cut), animal 1 showed a partial recovery in burst number from one to five, whereas animal 2 (20 h after cut) showed no recovery.

Overall, the number of swim cycles decreased significantly from 4.3 ± 1.0 to 2.8 ± 1.2 cycles (*N* = 87) immediately (<1 h) after PdN6 disconnection and then recovered back to 3.5 ± 1.2 cycles (*N* = 51). The data from the recovery period (10–30 h) could be separated into the following two groups: “well recovered” preparations, which increased the number of bursts by >25% (Fig. 3*B*); and “poorly recovered” preparations, which increased the number of bursts by <25% (Fig. 3*C*). Of the 51 preparations tested, 45.1% (*N* = 23) were well recovered preparations and 54.9% (*N* = 28) were poorly recovered preparations. Eleven of the poorly recovered preparations decreased, rather than increased in burst number over the course of 10–30 h. In well recovered preparations, the recovered cycle numbers at 10–20 and 20–30 h were both significantly greater than the impaired cycle numbers (Fig. 3*B*). Plots of binned data from well recovered preparations show that, after the initial decrease following the lesion, the number of swim cycles started to recover in 2–3 h, and by 5 h the number of swim cycles reached a level that was not significantly different from the control (Fig. 3*B*). In contrast, in those preparations that showed no recovery, the number of swim cycles stayed at a significantly lower level than the prelesion value for up to 30 h (Fig. 3*C*). Altogether, the CoV increased after the lesion and remained high for 30 h (Fig. 3*D*).

**Figure 4.**
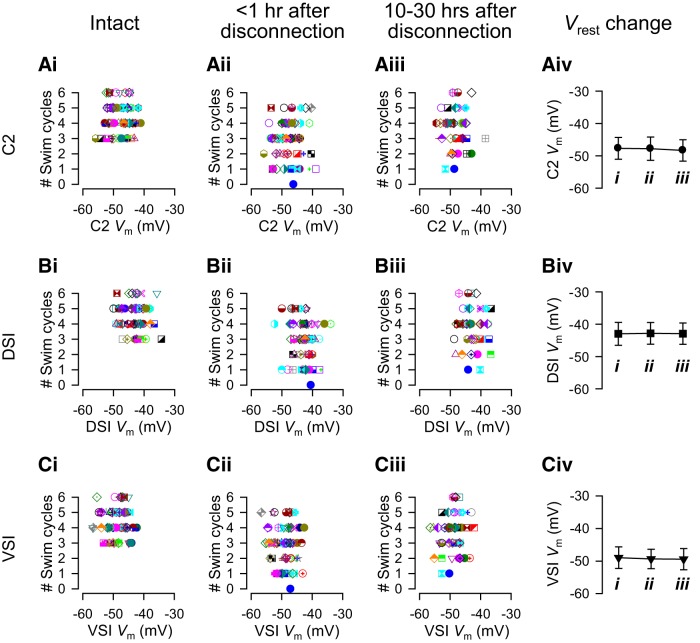
The number of swim cycles showed no correlation with the resting membrane potentials of the CPG neurons. ***Ai***–***iii***, ***Bi***–***iii***, ***Ci***–***iii***, The resting potential of C2 (***Ai***–***iii***), DSI (***Bi***–***iii***), and VSI (***Ci***–***iii***) showed no correlation with the number of swim cycles, and no change before and after PdN6 disconnection. With PdN6 intact, the mean resting membrane potentials for C2, DSI, and VSI were 47.7 ± 3.4, 43.0 ± 3.6, and 49.0 ± 3.3 mV, respectively (mean ± SD; *N* = 75, 67, and 92). PdN6 disconnection caused no significant change in their resting potentials (***Aiv***, ***Biv***, ***Civ***; *p* > 0.5 by one-way ANOVA). Colored graph symbols in Figures 4, 5, 8, 9, 10 and 11 each represent data from the same individuals.

There was no correlation between the number of swim cycles and the resting potential of the CPG neurons throughout the experiment (Fig. 4*Ai–iii*,*Bi–iii*,*Ci–iii*). Furthermore, there was no significant change in the resting potential over the course of the experiments (Fig. 4*Aiv*,*Biv*,*Civ*).

**Figure 5.**
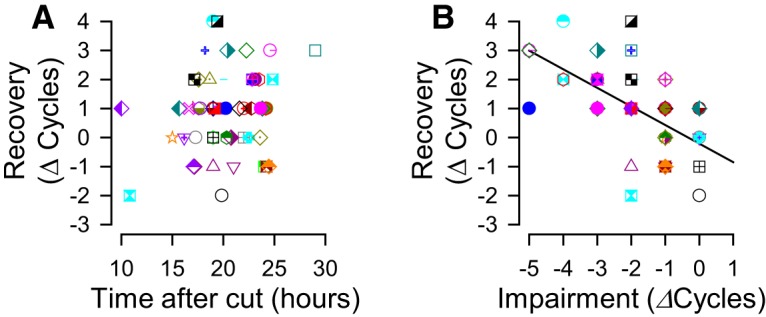
The extent of recovery was independent of time, but was dependent on the decrease in the number of swim cycles after cutting PdN6. ***A***, There is no significant correlation between the change in the number of swim cycles in isolated brain preparations and the recorded times after PdN6 disconnection (*p* = 0.13 by Pearson product moment correlation, *N* = 54). ***B***, The changes in the number of swim cycles after recovery was significantly correlated to the number of lost swim cycles caused by PdN6 disconnection (*R* ^2^ = 0.36, *p* < 0.001 by Pearson product moment correlation, *N* = 54).

The recovery process occurred within 10 h after PdN6 transection (Fig. 3), and beyond 10 h there was no correlation of recovery with time (Fig. 5*A*); rather, recovery was correlated with the extent of impairment (Fig. 5*B*). These results indicate that individual differences in the extent of recovery were not due to the differences in the times at which the measurements were made, and that the increased number of swim cycles the next day was the recovery from an impaired state and not a general time-dependent increase.

### PdN6 disconnection revealed the proximal synaptic action by eliminating the distal synapses

**Figure 6.**
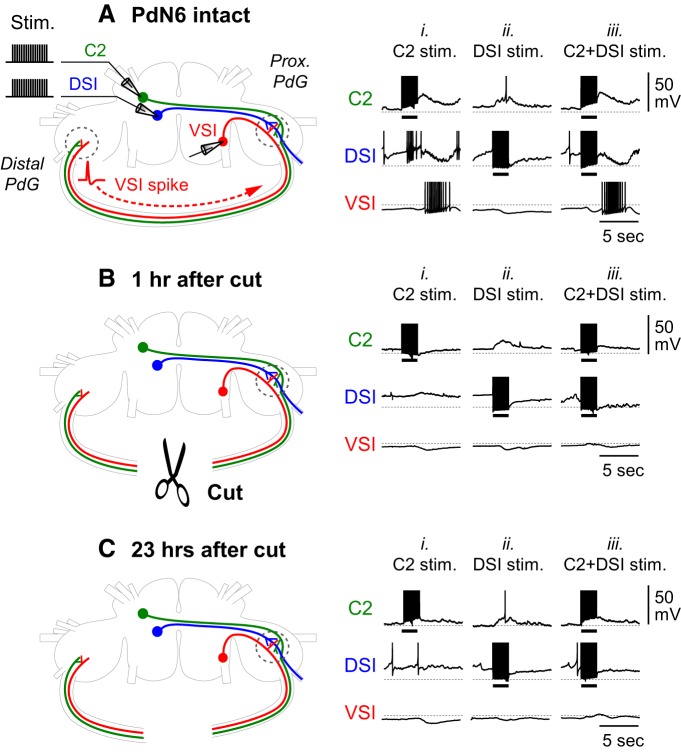
PdN6 disconnection impairs synaptic actions of C2 onto VSI. ***A***–***C***, Schematic drawings of a dorsal view of the *Tritonia* brain with the three CPG neurons (left) and simultaneously recorded activity of the three CPG neurons, while stimulating DSI and/or C2 (right) are shown. For stimulation, repetitive current pulses (8 nA, 20 ms) were injected into C2 or DSI, or both, at 10 Hz for 2 s while recording the membrane potential responses from VSI. ***A***, In an intact brain, C2 makes synaptic connections onto VSI in both pedal ganglia, as indicated by dotted circles. Stimulation of C2 (***Ai***), or C2 and DSI together (***Aiii***), evoked a burst of action potentials in VSI. It was previously shown that VSI spikes are generated in the distal pedal ganglion (PdG), and they propagate antidromically to the cell body, where the intracellular recording was made (Sakurai and Katz, 2009; Sakurai et al., 2014). Stimulation of DSI depolarized C2 and hyperpolarized VSI (***Aii***). Although these synaptic interactions among the CPG neurons were determined to be monosynaptic, unitary synaptic potentials corresponding one to one to the presynaptic spikes have never been recorded (Getting, 1981, 1983). ***B***, ***C***, After PdN6 disconnection, the distal synaptic action was eliminated, and C2 stimulation mainly produced a hyperpolarizing response in VSI in the proximal pedal ganglion (***Bi***, ***Ci***). The effect of DSI showed little change after PdN6 disconnection (***Bii***, ***Cii***). Although C2 and DSI stimulation both had inhibitory actions onto VSI, the combination of both (C2+DSI stim.) produced a slightly larger depolarizing component than C2 alone (***Biii***), which grew larger in the next day (***Ciii***). A dotted line on each trace indicates −50 mV membrane potential. All recordings were obtained in normal saline from the same preparation.

C2-evoked excitation of VSI is essential for production of the swim motor pattern (Getting, 1983; Calin-Jageman et al., 2007; Sakurai and Katz, 2009). With PdN6 intact, C2 stimulation (10 Hz for 2 s) induces a delayed burst of action potentials in VSI (Fig. 6*Ai*; Getting, 1983). It was previously shown that in most preparations (>80%), C2 excites VSI mainly in the distal pedal ganglion and consequently causes antidromic spikes that travel backward to the soma through PdN6 (Fig. 6*A*, schematic; Sakurai and Katz, 2009; Sakurai et al., 2014). Thus, when PdN6 is intact, the synaptic action of C2 in the proximal pedal ganglion is obscured by these antidromic spikes (Fig. 6*Ai*). Disconnection of PdN6 eliminates the distal synaptic action and reveals the proximal synaptic action of C2 onto VSI (Fig. 6*Bi*; Sakurai and Katz, 2009; Sakurai et al., 2014). C2 stimulation also caused a brief excitation in DSI when PdN6 was intact (Getting, 1981; Frost and Katz, 1996); however, it was eliminated after PdN6 disconnection. In this study, we did not further investigate the interaction between C2 and DSI, but focused on their synaptic actions onto VSI.

DSI causes VSI to hyperpolarize (Fig. 6*Aii*; Getting, 1981, 1983), which was not substantially altered after PdN6 disconnection (Fig. 6*Bii*). In this study, we also examined the effect of combined stimulation of C2 and DSI (C2+DSI stimulation), mimicking their activity during the swim motor pattern. Before PdN6 disconnection, C2+DSI stimulation evoked a burst in VSI (Fig. 6*Aiii*; 14.5 ± 12.3 spikes; *N* = 25), which is similar to C2 stimulation alone (Fig. 6*Ai*; 14.1 ± 11.5 spikes; *N* = 25; *p* = 0.90 by Student’s *t* test). As in C2 stimulation alone, the excitatory effect of C2+DSI stimulation on VSI was also largely diminished after PdN6 disconnection (Fig. 6*Biii*,*Ciii*).

### Synaptic actions of C2 and DSI change during recovery

**Figure 7.**
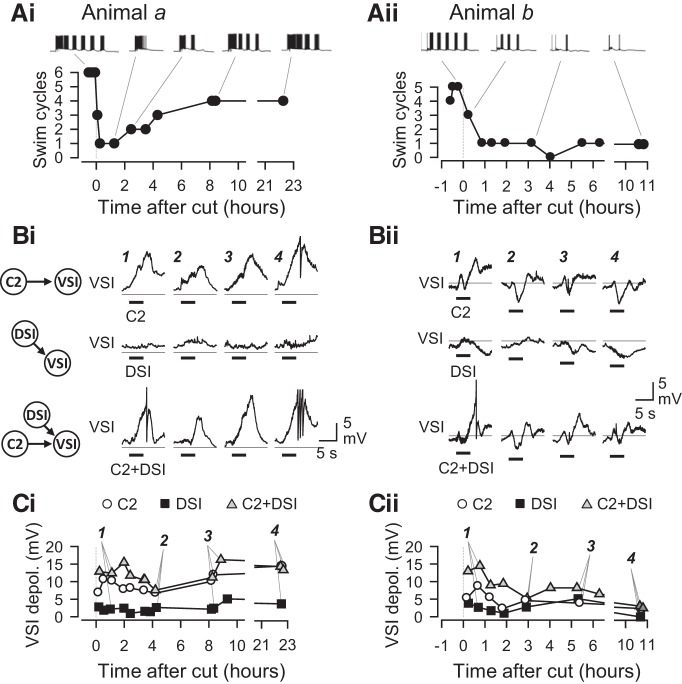
Time courses of changes in swim cycle and synaptic actions. Two examples showing temporal changes in the number of swim cycles together with the magnitudes of synaptic actions from C2 and/or DSI onto VSI. All recordings were made in normal saline. ***A***, Plots show the time courses of changes in the number of swim cycles recorded from two different preparations, animal a (***Ai***) and animal b (***Aii***). Example traces of VSI bursts in the swim motor pattern are shown above each plot. In the brain from animal a, the number of swim cycles dropped down from 6 to 1 cycle; but it recovered back to four cycles at 8 h after PdN6 disconnection. In animal b, the number of swim cycles never recovered. ***B***, Temporal changes in the synaptic actions within the CPG. Traces show membrane potential responses of VSI to C2 stimulation (top traces), DSI stimulation (middle traces), and combined stimulation of C2 and DSI (C2+DSI; bottom traces) in the brains from animal a (***Bi***) and animal b (***Bii***). The synaptic responses contain both monosynaptic and polysynaptic actions. In these examples, the polysynaptic component caused VSI to be depolarized even by DSI, which makes an inhibitory synapse onto VSI (Fig. 1*A*). C2 and C2+DSI stimulation produced monotonic depolarization in animal a, but they produced more complex waveforms with a distinctive hyperpolarization phase in animal b. Their waveforms were not stable but constantly changed over time. The number (1–4) above each trace in ***B*** corresponds to the numbered data point in ***C***. A horizontal line on each trace indicates a −50 mV level. ***C***, Graphs showing changes in the amplitudes of synaptic responses in VSI evoked by the stimulation of C2 (open circles), DSI (filled squares), or C2+DSI (gray triangles). In the brain from animal a, C2 and C2+DSI produced a large depolarization in VSI, which later increased in amplitude (***Ci***). In contrast, synaptic responses all decreased over time in the brain from animal b (***Cii***).

To determine the mechanism underlying the recovery of swim cycles, we looked for corresponding changes in the synaptic actions upon recovery. The synaptic actions of C2 and/or DSI and the swim motor pattern were recorded repeatedly at various times after PdN6 disconnection (Fig. 7). The synaptic actions of C2 and DSI changed in shape and amplitude throughout the time of recording. The time course of changes also differed among individuals. Figure 7 shows two examples of measurements of the swim motor pattern and synaptic actions recorded from different preparations (animals a and b). In animal a, the number of swim cycles dropped from six to three after PdN6 disconnection, and then down to one; but it showed a partial recovery back to four cycles in 8 h (Fig. 7*Ai*). In animal b, within an hour after PdN6 disconnection, the number of swim cycles dropped from five to one and showed no recovery, staying at one or zero for >10 h (Fig. 7*Aii*).

The synaptic actions of C2 and DSI in these two preparations evolved differently during recovery (Fig. 7*B*,*C*). In animal a, the stimulation of C2 (10 Hz for 4 s) produced a depolarizing potential in VSI, whereas DSI stimulation caused a very small response (Fig. 7*Bi*). C2+DSI stimulation evoked a depolarizing potential that was similar to, but slightly larger than, the effect of C2 alone. These VSI responses changed in amplitude over time after PdN6 disconnection, becoming smaller initially and then growing larger later (Fig. 7*Ci*). The C2^−^ and C2^+^ DSI-evoked potentials in VSI maintained their amplitude the next day. In contrast, in animal b, C2-evoked and DSI-evoked potentials had a mixture of both depolarizing and hyperpolarizing components (Fig. 7*Bii*); they showed somewhat complex changes but became smaller in amplitude by 10 h (Fig. 7*Cii*).

### The extent of recovery correlated with a change in the synaptic actions of C2 and DSI

**Figure 8.**
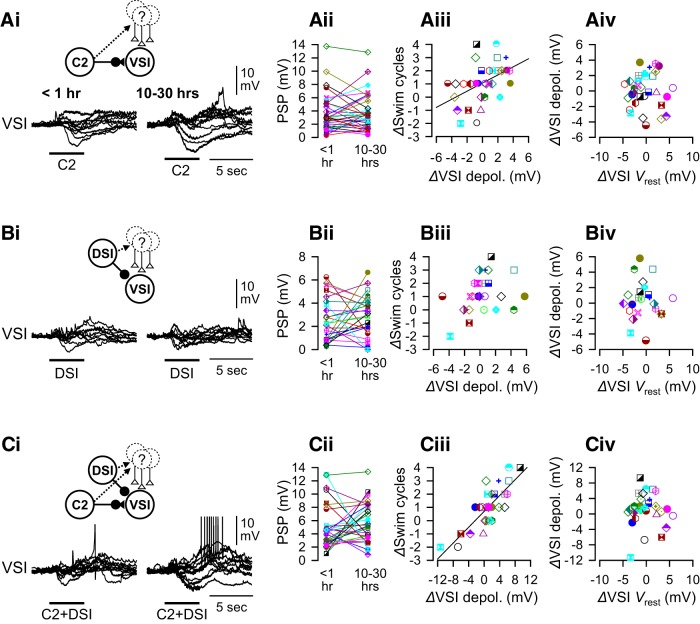
Changes in the extent of swim recovery correlated with polysynaptic actions. The amplitudes of synaptic actions of C2 and/or DSI onto VSI varied among individuals. ***A***, C2-evoked responses in VSI. Overlaid traces of C2-evoked synaptic responses in VSI (***Ai***) and plots of their amplitude (***Aii***) are shown. The recordings were made <1 h (left) and 10–30 h (right) after PdN6 disconnection. There is a significant positive correlation between changes in the number of swim cycles 10–30 h after PdN6 disconnection (*Δ*Swim cycles) and changes in the amplitudes of C2-evoked depolarization (*Δ*VSI depolarization by C2; *R* ^2^ = 0.20, *p* = 0.008 by a linear regression, *N* = 33; ***Aiii***). The change in the resting potential of VSI showed no correlation with the change in the amplitudes of C2-evoked depolarization (***Aiv***). ***B***, DSI-evoked synaptic responses in VSI. Overlaid traces of DSI-evoked responses (***Bi***) and plots of their amplitudes (***Bii***) at two different times were shown. No correlation was seen between changes in the number of swim cycles and the DSI synaptic action (***Biii***; *p* = 0.10 by a linear regression, *N* = 25). The change in the resting potential of VSI showed no correlation with the DSI-evoked depolarization (***Biv***). ***C***, Synaptic responses of VSI to the combined stimulation of C2 and DSI (C2+DSI). Overlaid traces of the responses (***Ci***) and plots of their amplitudes (***Cii***) at two different times were shown. There is a strong positive correlation between these parameters (***Ciii***; *R* ^2^ = 0.67, *p* < 0.001 by a linear regression, *N* = 30). The change in the resting potential of VSI showed no correlation with C2+DSI-evoked depolarization (***Civ***). The synaptic responses recorded in normal saline include both direct monosynaptic and polysynaptic actions, as indicated by insets above the overlaid traces. In ***Ai***, ***Bi***, and ***Ci***, overlaid traces from 10 representative preparations were shifted to align together at the onset of the synaptic potentials.

As shown previously, the response of VSI to the synaptic actions of other CPG neurons varied among individuals (Fig. 8; Sakurai et al., 2014). Shortly after PdN6 disconnection, stimulation of C2, DSI, or both (C2+DSI stimulation) caused complex membrane potential responses in VSI with both depolarizing and hyperpolarizing components (Fig. 8*Ai*,*Bi*,*Ci*, left overlaid traces). Since these recordings were made in normal saline, these postsynaptic responses include indirect, polysynaptic inputs mediated by unidentified neurons as well as direct synaptic action between the swim interneurons. In most preparations, C2+DSI stimulation produced a larger depolarization than those evoked by C2 or DSI alone and often induced spiking in VSI (Fig. 8*Ci*). 10 to 30 h after PdN6 disconnection, these polysynaptically evoked potentials showed a variety of changes across preparations (Fig. 8*Aii*,*Bii*,*Cii*, right overlaid traces).

The extent of recovery (*Δ*Swim cycles) showed a significant correlation with the change in C2-evoked polysynaptic depolarization (Fig. 8*Aiii*). The correlation became stronger with C2+DSI stimulation (Fig. 8*Ciii*). There was no correlation to changes in response to DSI alone (Fig. 8*Biii*). The changes in the amplitude of depolarization had no correlation with the changes in the resting potential of VSI (Fig. 8*Aiv*,*Biv*,*Civ*). Thus, the recovery of the swim motor program occurred together with the increase in the polysynaptic depolarization produced by the combined action of C2 and DSI synapses.

### The extent of recovery did not correlate with direct synaptic actions

**Figure 9.**
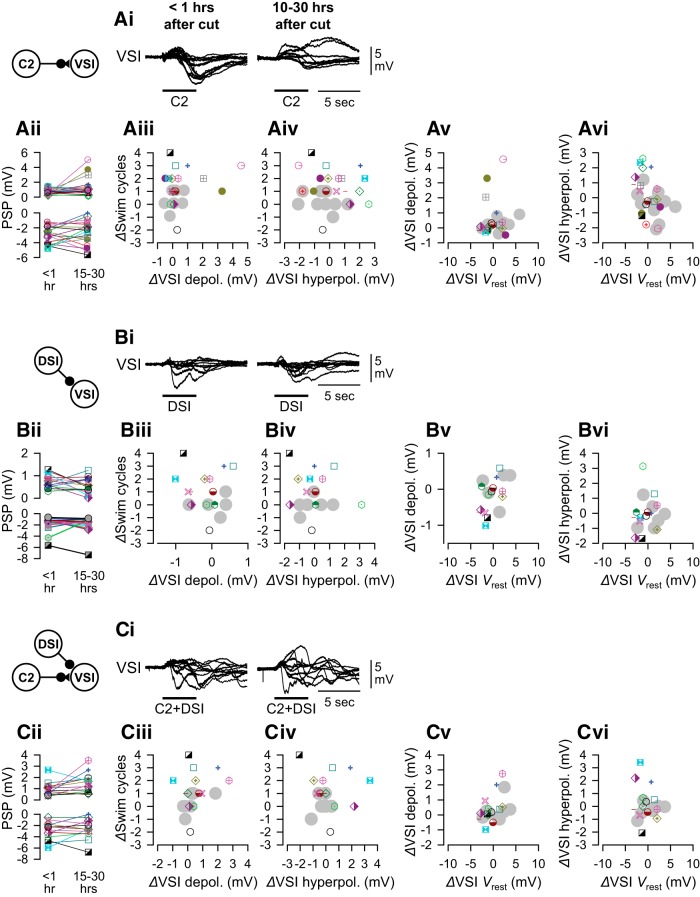
The direct synaptic actions of C2 and/or DSI onto VSI and their changes showed wide variations, but had little or no correlation with the changes in the number of swim cycles. All recordings were made in Hi-Di saline to minimize polysynaptic components. ***A***, C2-evoked responses in VSI. Overlaid traces of C2-evoked synaptic responses in VSI (***Ai***) and plots of their amplitudes of evoked depolarization (***Aii***, top) and hyperpolarization (***Aii***, bottom) are shown. Recordings were made <1 h (left) and 10–30 h (right) after the disconnection. C2 was stimulated by injecting repetitive current pulses (20 ms in duration at 10 Hz for 4 s). No correlation was seen between changes in swim cycles (*Δ*Swim cycles) and the amplitudes of C2-evoked depolarization (*Δ*VSI depolarization by C2; *p* = 0.43 by linear regression, *N* = 18; ***Aiii***) or C2-evoked hyperpolarization (*Δ*hyperpolarization by C2; *p* = 0.44 by linear regression, *N* = 18; ***Aiv***). The change in the resting potential of VSI showed no correlation with C2-evoked depolarization (***Av***) or hyperpolarization (***Avi***). ***B***, DSI-evoked synaptic responses in VSI. Overlaid traces of DSI-evoked responses (***Bi***) and plots of their amplitudes (***Bii***) at two different times were shown. No correlation was seen between changes in swim cycles and DSI-to-VSI synaptic action (***Biii*** and ***Biv***; *p* = 0.99 and 0.57 by linear regression, *N* = 13). The change in the resting potential of VSI showed no correlation with DSI-evoked depolarization (***Bv***) or hyperpolarization (***Bvi***). ***C***, Synaptic response of VSI to the combined C2+DSI stimulation. Overlaid traces of the responses (***Ci***) and plots of their amplitudes (***Cii***) at two different times were shown. No correlation was seen between changes in swim cycles and the synaptic actions (***Ciii*** and ***Civ***; *p* = 0.51 and 0.57 by linear regression, *N* = 13). The change in the resting potential of VSI showed no correlation with C2+DSI-evoked depolarization (***Cv***) or hyperpolarization (***Cvi***). Gray circles represent control data with no PdN6 disconnection. In ***Ai***, ***Bi***, and ***Ci***, overlaid traces from 10 representative preparations were shifted to align at the onset of the synaptic potential.

Functional recovery of the swim circuit was previously suggested to occur through enhanced monosynaptic excitation of VSI by C2 (Sakurai and Katz, 2009). Moreover, it was recently shown that the degree of reduction in the number of swim cycles after cutting PdN6 was highly dependent upon the amplitude of C2-evoked hyperpolarization (Sakurai et al., 2014). Therefore, we examined the relationship between the extent of recovery in the number of swim cycles and the change in the direct synaptic actions of both C2 and DSI onto VSI (Fig. 9).

To examine the direct synaptic actions of C2 and DSI, Hi-Di saline was used to raise the threshold for action potential firing and consequently minimized polysynaptic actions. Although these synaptic actions are likely monosynaptic, neither C2 nor DSI stimulation evoked unitary synaptic potentials corresponding one to one to the presynaptic spikes (Getting, 1981, 1983). Rather, the stimulation of C2 produced complex slow postsynaptic responses in VSI, with an initial small depolarization and a subsequent large inhibitory component, which varied across individuals (Fig. 9*Ai*, left). These waveforms changed by the next day (Fig. 9*Ai*, right). In some preparations, C2 produced a notably strong depolarization in VSI with a greatly reduced inhibitory component (Fig. 9*Ai*, right). DSI produced a hyperpolarizing response in most preparations (25 in 26 preparations), but the waveform was variable among individuals (Fig. 9*Bi*). Combined stimulation of C2 and DSI together produced VSI responses that appeared similar to the response evoked by C2 alone in 50% of preparations (14 in 28 preparations), but in others such stimulation produced complex subthreshold oscillatory responses with small depolarizations and hyperpolarizations (14 in 28 preparations; Fig. 9*Ci*). This was particularly true the day after cutting PdN6. In all cases, the synaptic responses in VSI changed in amplitude and/or waveform the day after PdN6 disconnection and were highly variable among preparations (Fig. 9*Aii*,*Bii*,*Cii*). However, the extent of recovery did not correlate with any of these changes in synaptic actions (Fig. 9*Aiii*,*Aiv*,*Biii*,*Biv*,*Ciii*,*Civ*). These changes in synaptic actions showed no correlation with the resting potential of VSI (Fig. 9*Av*,*Avi*,*Bv*,*Bvi*,*Cv*,*Cvi*).

### Recovery of the swim motor pattern correlated with recruitment of polysynaptic pathways

**Figure 10.**
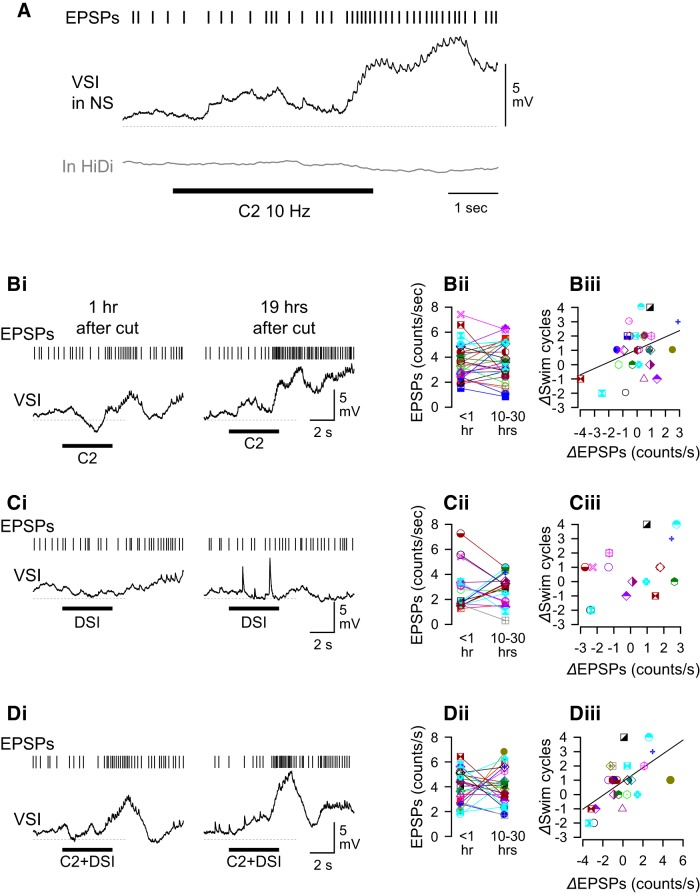
The recovery in the number of swim cycles was correlated with the change in the recruitment of polysynaptic inputs by C2 stimulation alone or by C2 and DSI stimulated together. ***A***, C2 stimulation (black bar) caused a delayed increase in the number of small EPSPs recorded in VSI from unknown sources in normal saline (black trace). C2 was stimulated with repeated current pulses (20 ms, 10 nA at 10 Hz) for 4 s. Vertical bars above the voltage trace indicate the times of small EPSPs. Only EPSPs >0.1 mV were counted. The small EPSPs disappeared when Hi-Di saline was applied (gray trace). A dotted line indicates −50 mV level. ***Bi***, Examples of VSI membrane potential traces in response to C2 stimulation at two different times (left, 1 h; right, 19 h after PdN6 transection). Tick marks above each trace indicate the times of small EPSPs that rode on top of basal membrane fluctuation in VSI. C2 stimulation caused an increase in the frequency of the small EPSPs. The recruitment of EPSPs increased 19 h after PdN6 transection. ***Bii***, Plot of the EPSP frequencies at two times show individual variations in the direction of changes in their frequencies. EPSP frequency was measured in a 6 s window after the C2 stimulation. ***Biii***, The change in the number of swim cycles (*Δ*Swim cycles) 10–30 h after PdN6 disconnection showed a weak but significant correlation with the change in C2 recruitment of EPSPs (*R* ^2^ = 0.15, *p* = 0.04 by linear regression, *N* = 28). ***C***, Stimulation of DSI slightly increased the occurrence of spontaneous EPSPs in VSI. ***Ci***, An example of DSI-evoked membrane potential responses in VSI at two different times and the occurrence of small EPSPs. ***Cii***, Plot of the EPSP frequencies at two times shows individual variations in the direction of changes in their frequency. ***Ciii***, The change in the swim cycles showed no significant correlation to the change in frequency of DSI-evoked EPSPs (*R* ^2^ = 0.15, *p* = 0.11 by linear regression, *N* = 18). ***D***, Stimulation of C2 and DSI together increased the appearance of spontaneous EPSPs in VSI. ***Di***, An example of VSI membrane potential responses and recruitment of small EPSPs after a combined stimulation of C2 and DSI at two different times. ***Dii***, Plot of the EPSP frequencies at two times show individual variations in the direction of changes in their frequency. ***Diii***, The change in swim cycles (*Δ*Swim cycles) 10–30 h after PdN6 disconnection shows a strong correlation with the increase in EPSP frequency (*R* ^2^ = 0.36, *p* = 0.002 by linear regression, *N* = 23). A dotted line under each trace indicates −50 mV membrane potential.

Previously, it was reported that C2 stimulation in normal saline recruits a bombardment of small EPSPs in VSI that did not correspond one to one with presynaptic C2 spikes, indicating that these membrane potential responses contained both monosynaptic and polysynaptic components (Fig. 10*A*,*Bi*). The increase of EPSP frequency occurs during or after the stimulation and lasted for 3–15 s. The overall depolarization produced by the recruited EPSPs has little role in exciting VSI when PdN6 is intact but plays a large part in causing the excitation of VSI after PdN6 disconnection (Sakurai et al., 2014). Here we found that similar EPSP bombardments were also induced by the stimulation of DSI, or by the combined stimulation of C2 and DSI together (Fig. 10*Ci*,*Di*).

The extent of changes in the number of recruited EPSPs varied widely across preparations (Fig. 10*Bii*,*Cii*,*Dii*). The extent of recovery (*Δ*Swim cycles) showed a weak but significant correlation with the changes in the rate of EPSPs recruited by C2 stimulation (Fig. 10*Biii*). DSI alone caused a variety of changes in the EPSP frequency, but they did not correlate with the extent of recovery (Fig. 10*Ciii*). When the stimulation of C2 and DSI were combined, a stronger correlation was seen between the changes in the rate of recruited EPSPs and the extent of recovery (Fig. 10*Diii*). Together, these results suggest that the recovery of the swim motor pattern after PdN6 disconnection was not likely due to changes in the strength of direct synapses within the swim CPG circuit but, rather, involves changes in polysynaptic actions, especially those mediated by C2 and DSI together through unidentified neurons outside of the canonical CPG circuitry.

### Synaptic recruitment contributed to intraburst VSI spiking

**Figure 11.**
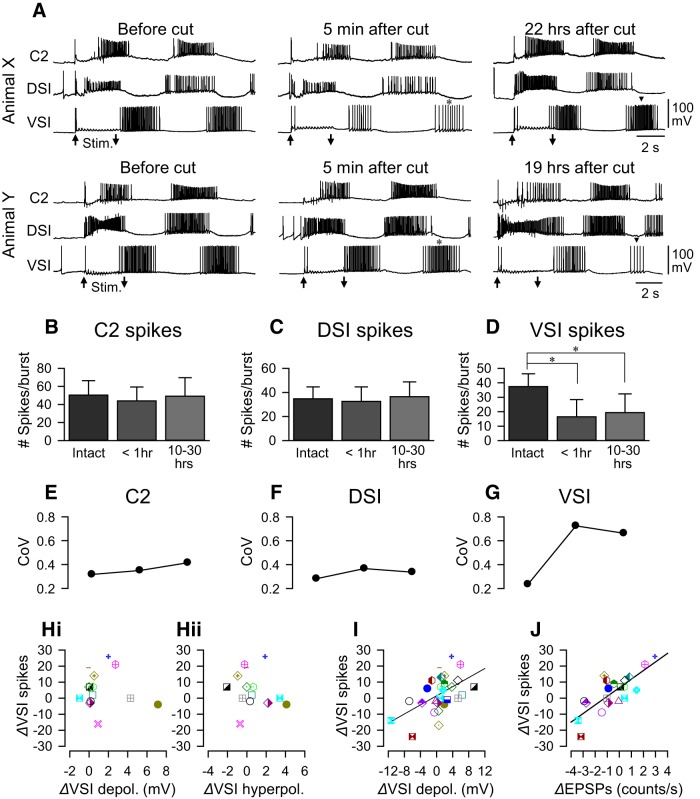
The change in the intraburst spike rate in VSI correlated with the extent of polysynaptic actions induced by combined action of C2 and DSI. ***A***, Simultaneous intracellular recordings from C2, DSI, and VSI from two representative animals (animals X and Y). Five minutes after cutting PdN6, animal X showed a decrease in the number of VSI spikes during the second burst of the swim motor pattern (asterisk). In contrast, there was little effect on the spiking in the second VSI burst in animal Y (asterisk). Twenty-two hours after PdN6 cut, VSI spiking during the second burst of the swim motor pattern recovered in animal X (arrowhead), whereas 19 h after PdN6 the VSI in animal Y fired fewer spikes than before (arrowhead). Arrows (Stim) indicate the time of PdN3 stimulation. ***B***–***D***, Bar graphs show the average number of spikes of C2 (***B***), DSI (***C***), and VSI (***D***) in the second burst in the swim motor pattern counted before cutting PdN6 (Intact), shortly after cutting PdN6 (<1 h), and the next day (10–30 h). Before cutting PdN6, the number of spikes in the second bursts were 50.4 ± 16.0 (C2, *N* = 45), 34.8 ± 9.8 (DSI, *N* = 41), and 37.5 ± 8.8 (VSI, *N* = 45). Shortly after the cut (<1 h), they were 44.0 ± 15.4 (C2, *N* = 43), 32.7 ± 12.0 (DSI, *N* = 39), and 16.5 ± 12.0 (VSI, *N* = 47). The next day (10–30 h), they became 49.3 ± 20.5 (C2, *N* = 36), 36.5 ± 12.3 (DSI, *N* = 30), and 19.5 ± 12.9 (VSI, *N* = 41). The average number of VSI spikes shortly after cutting PdN6 and the next day were significantly lower than before the cut, as indicated by asterisks (*p* < 0.001 by Kruskal–Wallis one-way ANOVA on ranks with Dunn’s method). ***E***–***G***, Plots of CoV corresponding to the bar graphs above (***B***–***D***). C2 and DSI showed relatively constant variance throughout the experiments (***E***, ***F***), whereas VSI showed an increase in variance (***G***). There was a significant difference in variance of the VSI spike number between intact and <1 h (*p* = 0.02 by Levene median test) and between intact and 10–30 h (*p* = 0.002 by Levene median test). ***H***, C2-evoked direct synaptic responses in VSI showed no correlation with the changes in the number of VSI spikes in the second burst of swim motor patterns. Changes in the number of VSI spikes in the second burst of motor patterns 15–30 h after PdN6 disconnection (*Δ*VSI spikes) were plotted against the change in the amplitudes of the depolarization phase of the C2+DSI-evoked synaptic potential measured (*Δ*VSI depolarization, ***Hi***) or that of the C2+DSI-evoked hyperpolarization phase (Δhyperpolarization, ***Hii***) in Hi-Di saline. There was no correlation between these parameters (*p* = 0.78 and 0.57, *N* = 14). ***I***, The changes in the intraburst VSI spikes (*Δ*VSI spikes) upon recovery showed a significant correlation with the changes in the polysynaptic depolarization in VSI evoked by C2+DSI stimulation in normal saline (*R* ^2^ = 0.28, *p* = 0.004 by linear regression, *N* = 27). ***J***, The changes in the number of VSI spikes in the second burst upon recovery was correlated with the changes in the frequency of small EPSPs recruited by C2+DSI stimulation (*R* ^2^ = 0.63, *p* < 0.001 by linear regression, *N* = 20).

Along with the changes in the number of swim cycles, the number of spikes in each VSI burst also changed upon PdN6 disconnection and after the recovery. As with the other measures, there was a large degree of individual variability in spiking. Figure 11*A* shows examples of the first two bursts in DSI, C2, and VSI recorded from two different preparations (animal X and animal Y). Shortly after cutting PdN6 (5 min after cut), a remarkable decrease in the number of VSI spikes in the second burst was seen in animal X, whereas less of a change was seen in animal Y (Fig. 11*A*, asterisks). The next day (22 h after cut), the intraburst VSI spikes showed a recovery in animal X (arrowhead), whereas in animal Y the VSI showed a substantial decrease in the second burst (arrowhead, 19 h after cut). On average, the number of intraburst spikes in DSI and C2 showed no significant changes (*p* = 0.38 and 0.17 by one-way ANOVA; Fig. 11*B*,*C*), and there were only small changes in the coefficient of variance throughout the experiment (Fig. 11*E*,*F*). In contrast, VSI showed a significant decrease in the average number of spikes in the second burst after PdN6 disconnection (Fig. 11*D*). The intraburst spike number of VSI remained significantly lower than control in the next day (10–30 h). The coefficient of variance showed a steep increase after disconnecting PdN6 and stayed high in the next day (Fig. 11*G*).

The changes in the number of intraburst VSI spikes 10–30 h after PdN6 disconnection showed no correlation with the changes in the direct synaptic actions of C2 and DSI recorded in Hi-Di saline (Fig. 11*Hi*,*Hii*). They were significantly correlated with the changes in the amplitude of the polysynaptic depolarization evoked in VSI by C2+DSI stimulation (Fig. 11*I*) and with the change in the frequency of EPSPs recruited by C2+DSI stimulation (Fig. 11*J*). Thus, the results suggest that the extent of changes in the intraburst VSI spikes was also under a strong influence of the changes in the polysynaptic actions mediated by C2 and DSI bursts.

## Discussion

In this study, the effect of a lesion to a neuronal circuit for the escape swim behavior of a nudibranch *T. diomedea* was examined. There were animal-to-animal differences in the ability of the behavior to recover after severing a commissure that connects the two pedal ganglia, thereby removing some swim CPG synapses. Here, we measured the change in the efficacy of excitatory synaptic connections, both direct and indirect, between individual neurons that are major constituents of the swim CPG. Individual differences in recovery correlated with changes in the extent to which CPG neurons recruited polysynaptic input onto other CPG neurons.

### Synaptic recruitment underlies the functional recovery of the CPG

**Figure 12.**
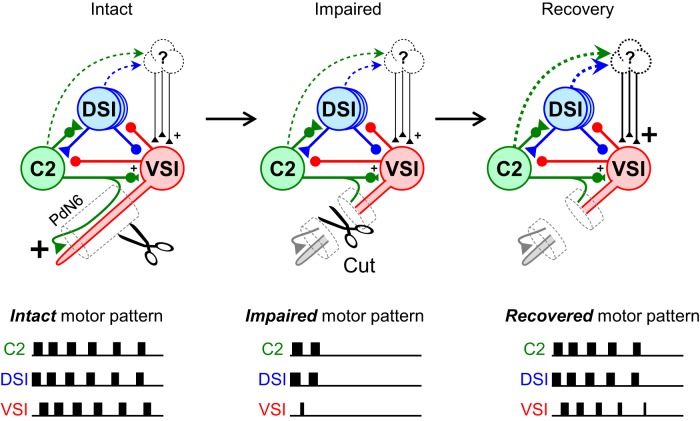
Mechanism underlying the functional recovery of the swim motor circuit after a partial lesion of the network. C2 synapses onto VSI in at least two places, one proximal and the other distal to PdN6. C2 and DSI both make indirect excitatory inputs (dashed lines) onto VSI through unidentified neurons. Under normal conditions, C2 excites VSI mainly through the excitatory synapse at the distal site (Sakurai and Katz, 2009; Sakurai et al., 2014), while it also recruits polysynaptic pathways (Sakurai et al., 2014). Upon disconnecting PdN6, the distal excitatory synapse from C2 to VSI is lost (middle). The swim motor pattern is often impaired because VSI receives less excitation. Upon recovery, polysynaptic pathways involving the recruitment of unidentified neurons play a larger role in providing excitation from C2 to VSI. The size of the "+" signs indicates the strength of excitatory synaptic inputs onto VSI.

It was not the direct C2-to-VSI synapse but the enhanced polysynaptic recruitment of excitatory inputs that played a major role in the recovery of swim cycles. Modeling studies previously showed that C2-evoked excitation of VSI is critical for producing the bursting pattern of activity (Getting, 1989a; Calin-Jageman et al., 2007). A lesion of PdN6 causes a significant loss of excitation from C2 to VSI, and this is the direct cause of the decrease in the number of swim cycles (Sakurai and Katz, 2009; Sakurai et al., 2014). To recover the ability to generate the motor pattern, C2 needs to regain its excitatory action onto VSI. Previously, Sakurai and Katz (2009) suggested that a change in the valence of the C2 synapse from primarily inhibitory to excitatory mediates the functional recovery of the swim circuit. Indeed, the direct C2-to-VSI synaptic potential showed a remarkable increase in some preparations (Figs. 9*Ai*,*Aii*); however, there were no correlations between the changes in the amplitude of C2/DSI-evoked synaptic potentials and the extent of swim recovery (Fig. 9). In contrast, the changes in the polysynaptic actions of C2, or the combination of both C2 and DSI, onto VSI all showed significant correlations with the extent of recovery in the number of swim cycles and the number of intraburst VSI spikes (Figs. 8, 11). Moreover, both of those measures showed significant correlations with the changes in the frequencies of recruited EPSPs in VSI by C2 or C2+DSI stimulation (Figs. 10, 11). Thus, after the loss of synaptic excitation by PdN6 disconnection, reconfiguration of the neuronal network through changes in indirect polysynaptic connections played a major role in the recovery of the swim CPG function. The recruitment of excitatory synapses onto VSI by C2 and DSI is at least partially responsible for this functional recovery. (Fig. 12).

### Functional recovery in other systems

Studies on flatworms (Koopowitz et al., 1975; Faisst et al., 1980) and garden slugs (Broyles and Sokolove, 1978) suggested that the reorganization of neuronal networks that were not damaged underlies the functional recovery of behaviors after a partial lesion of the nervous system. In the crustacean stomatogastric ganglion, it has been shown that decentralization caused by removing the extrinsic neuromodulatory inputs impairs the production of rhythmic activity, but a stable new activity pattern spontaneously recovers within hours to days (Thoby-Brisson and Simmers, 1998, 2002; Luther et al., 2003). Moreover, the long-term action of neuromodulatory inputs and the regulation of Ca^+^ and K^+^ conductances were suggested to underlie the spontaneous homeostatic recovery of oscillatory activity (Haedo and Golowasch, 2006; Zhang et al., 2009).

Similar reorganization occurs in mammalian nervous systems. It has long been suggested that entire cortical networks participate in the recovery process rather than local axonal regrowth in the damaged area (Carmichael, 2003; Nudo, 2006; Cramer, 2008a,b; Macias, 2008; Winship and Murphy, 2009). Functional recovery following spinal cord and peripheral nerve injury is caused by corticospinal reorganization at multiple levels of the motor system (Wall and Egger, 1971; Pons et al., 1991; Jain et al., 1997; Bruehlmeier et al., 1998; Green et al., 1998; Curt et al., 2002; Kim et al., 2006; Endo et al., 2007; Ghosh et al., 2009; Tandon et al., 2009; Ghosh et al., 2010; Oudega and Perez, 2012; Nardone et al., 2013). During recovery from spinal cord injury, the recruitment of new synapses or the enhancement of weak synapses was suggested to cause shifting in sensory–motor pathways in the spinal reflex (Martinez et al., 2011; Rossignol and Frigon, 2011; Nardone and Trinka, 2015). It has been suggested that the absence of input from higher sources may induce the reorganization of local neural circuitry that was affected by lesion (Dietz, 2010), which can be observed as a shifting of neuronal pathways that underlie sensory–motor reflexes (Hubli et al., 2011).

### The potential mechanisms of functional recovery

In this study, we could not determine the synaptic mechanism of the synaptic recruitment underlying the recovery. It is unknown which neurons were recruited by C2 and/or DSI to provide excitatory synapses onto VSI and how the combined action of C2 and DSI evoked a stronger excitation in VSI. All neurons in the swim CPG are bilaterally arranged and are electrically coupled to their contralateral counterparts (Getting, 1989b; Katz, 2009). Since the brain was twisted around the cerebral commissure to gain access to all three neurons in this study, it is still unknown whether the connections between two halves of the circuit played any role in the functional recovery. There is no evidence so far that C2 and/or DSI make synaptic connections with the ipsilateral VSI.

It is not clear how C2+DSI stimulation produced a stronger correlation with recovery than C2 stimulation alone. DSI and C2 may together activate a common target neuron that excites VSI; however, it seems less likely because the change in the synaptic recruitment evoked by DSI alone showed no correlation with the recovery. Another possibility is that the DSI modulates the strength of C2 synapses onto the recruited excitatory neuron. It was previously shown that the DSI is serotonergic and presynaptically enhances transmitter release from C2 (Katz et al., 1994; Katz and Frost, 1995; Sakurai and Katz, 2003). In mammals, serotonin has been shown to play a role in the recovery of spinal cord injury (Rossignol et al., 1998; Giroux et al., 1999; Antri et al., 2002, 2003,2005; Courtine et al., 2009). Even in the absence of serotonin, constitutive activity in serotonin receptors can restore large persistent calcium currents in motor neurons (Murray et al., 2010). This indicates that, in addition to the synaptic reconfiguration, changes in the magnitude of neuromodulatory actions by DSI may also play a role in the recovery of the swim circuit function. In this study, we did not investigate the contribution of serotonergic neuromodulation in the recovery process. Further studies are needed to address the role of neuromodulation in the functional recovery of a neural circuit.

### Fluid nature of the neuronal network

The functional recovery of the swim CPG occurred over a few hours. This is much faster than the recovery from injury in many other systems, which usually takes weeks to months (Cohen et al., 1986; Barbeau and Rossignol, 1987; Bareyre et al., 2004; Byrnes et al., 2009; Nudo, 2013; Takenobu et al., 2013; Harley et al., 2015). Moreover, both monosynaptic and polysynaptic connections not only differ among individuals, but they also changed constantly in efficacy over time after the lesion. This may indicate that the functional recovery of the motor pattern generation was mediated by plasticity in synapses changing autonomously over time.

Such a fluid nature in network structure has been suggested in mammals. Neuronal circuits are often able to maintain their consistent outputs in the face of perturbations (Turrigiano, 1999, 2011,2012). In the cortex and spinal cord, trial-to-trial variability in the size and/or pattern of the activated neuronal population has been reported (Shadlen and Newsome, 1998; Bair et al., 2001; Cai et al., 2006; Hansen et al., 2012). It was suggested that continuous changes in functional connectivity within the motor pools and in their activation patterns underlie such variability (Cai et al., 2006; Cramer, 2008a; Nudo, 2013). In mice, experimentally induced stroke reduced the response evoked by limb stimulation contralateral to the stroke, but it enhanced responses in the unaffected cortex to sensory stimulation of either contralateral or ipsilateral pathways within 30–50 min of stroke onset (Mohajerani et al., 2011). Immediate functional reorganization in the primary somatosensory cortex has been reported in rats after lesion to the spinal cord or a peripheral nerve (Aguilar et al., 2010; Humanes-Valera et al., 2013, 2014; Moxon et al., 2014; Yagüe et al., 2014). In the cat spinal cord, locomotor ability can be recovered quickly within 24 h after a complete spinalization if the animal had previously experienced a spinal hemisection (Barrière et al., 2008). It was suggested that spinal hemisection induces changes in the spinal circuitry, which brings the spinal circuitry to a primed state for re-establishing the locomotor circuit after complete spinalization.

It is common in both vertebrates and invertebrates that rhythmic circuits change network architecture even under normal conditions when there is a shift in the speed of the rhythm. For example, in vertebrate swimming circuits, more premotor interneurons are incorporated into the rhythmic activity as the swim frequency increases (Sillar and Roberts, 1993), or distinct sets of interneurons with different firing properties are selectively recruited (Li et al., 2007; McLean et al., 2007, 2008; Berkowitz et al., 2010; Ausborn et al., 2012). In the pteropod mollusc, *Clione limacina*, an increase in the swim cycle frequency is produced through the recruitment of interneurons (type 12 interneurons), which are silent at low swimming rates (Arshaversusky et al., 1985).

In *Tritonia*, trial-to-trial variation exists in neuronal activity even with no difference in behavioral output. Using a voltage-sensitive dye to record activity of large numbers of neurons outside of the canonical neuronal circuitry, it was shown that there was inconsistent activation of neurons (Hill et al., 2012). Moreover, sensitization after repetitive swim episodes recruits more neurons into rhythmic activity (Hill et al., 2015). It is not clear whether these changes occur in the CPG or in the population of efferent neurons. Our results indicate that similar malleable characteristics exist within the *Tritonia* swim CPG circuitry itself.

In the snail, *Lymnaea stagnalis*, specific synapses in the respiratory CPG constantly change in sign, indicating that even the valence of synaptic transmission can be modulated by environmental and neurohumoral conditions (Magoski and Bulloch, 2000). Similar continuous changes may occur in the *Tritonia* swim network which would alter the balance of polysynaptic excitation and monosynaptic inhibition between the CPG neurons, and, hence, may underlie flexibility and the robustness of the motor pattern generation.

### Triggers for reorganization

The signal that triggers and/or accelerates the adaptive recovery of the *Tritonia* swim CPG after the loss of axonal connections has yet to be determined. The swim motor program is episodic; C2 and VSI are silent until the swim motor program is evoked. It is not known how execution of the swim motor program affects the recovery process. Moreover, the number and the interval of swim episodes after the transection appeared to have little effect on the recovery (Fig. 2). Further studies are needed to identify those newly recruited neurons and the changes in their activity during the motor program before and after the recovery. This may provide a simple system to study general principles of how individuals are variable in the susceptibility to neural injury and in the ability to recover from the functional loss.

## Synthesis

The decision was a result of the Reviewing Editor Elizabeth Cropper and the peer reviewers coming together and discussing their recommendations until a consensus was reached. A fact-based synthesis statement explaining their decision and outlining what is needed to prepare a revision is listed below. The following reviewers agreed to reveal their identity: Karen Mesce, Rhanor Gillette.

The reviewers found that issues that were previously raised were addressed. Further concerns are minor (mainly typos) and are listed below:

Reviewer #1

The paper has improved and my concerns have been addressed. The paper could use some proofing--I did the best I could do. Not completely on board with the way that the summary cartoon is presented at the beginning of the paper. The cartoon is helpful but has no legend, and not sure if that will work for all. Authors need to remove the paragraph on p18 (lns 440-448) as it's really not relevant to the data presented.

111: was discarded if one of "the" neurons died during the recordings.

112: The swim motor program was evoked by stimulating "the" left PdN3 with a train of voltage.

120: It (remove has) was previously shown that no statistical difference between cutting and

122: The responses varied among preparations; however (add comma after however)

140: "The resting membrane potential"....

157: peak of "the" preceding depolarization...

181: and after "the" PdN6 disconnection test at two different test intervals.

184: the swimming behavior was tested at 8 hours and at 24 hours after "the" cut.

190-191: The change in the number of body flexion"s" (make pleural) 19-24 hours after "the" cut showed a significant correlation with the cut-induced reduction (of what? finish sentence)

203: at all after "the" PdN6 lesion as shown in Figure 2Cii.

205: the sham operated animal "were" from -1 to 5 cycles

225: 54.9% (N=28) were poor "ly"-recovered preparations

230: swim cycles started to recover in 2-3 hours (add comma here)"," and by 5 hours, (remove this comma here) the number of swim cycles reached

232: the number of swim cycles stayed at "a" significantly lower

235-236: There was no correlation between the number of swim cycles and the resting potential (OF WHAT?) throughout the experiment...(note: need specify which cells you're referring to

438: (Dietz, 2010), which can be observed as "a" shifting of neuronal pathways that underlie sensory

440-448: Delete this entire section; it has no relevance to the data presented and presents a distraction to the reader.

478: than the recovery from injury in many other systems, which usually take ("s") weeks to months

498: complete spinalization, (remove comma) if the animal had previously experienced a spinal hemisection

508: Clione limacine (add comma here) an increase in the swim cycle frequency is produced through the recruitment of

512-513: Using "a"voltage-sensitive dye to record activity of large numbers of neurons

outside of the canonical neuronal circuitry, it was shown that there "was" inconsistent activation of

528: episodic; C2 and VSI are silent until the swim motor program was (replace with "is" ) evoked.

652-653: A, Plots show the time courses of changes in the number of swim cycles in (remove in) recorded from two different preparations

664: "a" distinctive hyperpolarization phase in Animal b.

668: In the brain from Animal a, C2 and C2+DSI produced "a" large

676-677: their amplitude (Aii) in VSI recorded less than one hour after PdN6 disconnection and 10-30 hours the disconnection were shown (Re-write sentence).

796: C2 synapses onto VSI in at least two place"s",

Reviewer #2

The anaesthetic 1-phenoxy-2-propanol is mispelled in line 74.

I misunderstood the double-blind reason for adding "anonymous" as a citation in the text, but it still doesn't seem to be useful in any way at all. How could that citation be used by a reviewer to corroborate statements in the text?

I have no further questions about saline Na+ levels. Reviewing the salinity fluctuations at Friday Harbor for 5 years, I found that the saline values used are well within the normal range. The authors might be interested to privately compare their saline Na+ levels with that of Instant Ocean.
